# Maternal consumption of a high-fat diet modulates the inflammatory response in their offspring, mediated by the M1 muscarinic receptor

**DOI:** 10.3389/fimmu.2023.1273556

**Published:** 2023-12-18

**Authors:** Suleyma Oliveira Costa, Wenicios Ferreira Chaves, Priscilla Karla Fernandes Lopes, Iracema M. Silva, Beatriz Burguer, Leticia M. Ignácio-Souza, Adriana Souza Torsoni, Marciane Milanski, Hosana Gomes Rodrigues, Mina Desai, Michael Glenn Ross, Marcio Alberto Torsoni

**Affiliations:** ^1^ Laboratory of Metabolic Disorders, School of Applied Sciences, University of Campinas, Limeira, Brazil; ^2^ Laboratory of Nutrients and Tissue Repair, School of Applied Sciences, University of Campinas, Limeira, Brazil; ^3^ Obesity and Comorbidities Research Center, University of Campinas, Campinas, Brazil; ^4^ Department of Obstetrics and Gynecology, David Geffen School of Medicine, University of California Los Angeles at Harbor-UCLA, Torrance, CA, United States

**Keywords:** high fat diet (HFD), cholinergic, hypothalamus, obesity, muscarinic 1 acetylcholine receptors, DOHaD (Developmental origins of health and disease), maternal programming

## Abstract

**Introduction:**

High-fat diet (HFD) consumption is associated with various metabolic disorders and diseases. Both pre-pregnancy and maternal obesity can have long-term consequences on offspring health. Furthermore, consuming an HFD in adulthood significantly increases the risk of obesity and metabolic disorders. However, an intriguing phenomenon known as the obesity paradox suggests that obesity may confer a protective effect on mortality outcomes in sepsis. In sepsis, activation of the cholinergic anti-inflammatory pathway (CAP) can help mitigate systemic inflammation. We employed a metabolic programming model to explore the relationship between maternal HFD consumption and offspring response to sepsis.

**Methods:**

We fed female mice either a standard diet (SC) or an HFD during the pre-pregnancy, pregnancy, and lactation periods. Subsequently, we evaluated 28-day-old male offspring.

**Results:**

Notably, we discovered that offspring from HFD-fed dams (HFD-O) exhibited a higher survival rate compared with offspring from SC-fed dams (SC-O). Importantly, inhibition of the m1 muscarinic acetylcholine receptor (m1mAChR), involved in the CAP, in the hypothalamus abolished this protection. The expression of m1mAChR in the hypothalamus was higher in HFD-O at different ages, peaking on day 28. Treatment with an m1mAChR agonist could modulate the inflammatory response in peripheral tissues. Specifically, CAP activation was greater in the liver of HFD-O following agonist treatment. Interestingly, lipopolysaccharide (LPS) challenge failed to induce a more inflammatory state in HFD-O, in contrast to SC-O, and agonist treatment had no additional effect. Analysis of spleen immune cells revealed a distinct phenotype in HFD-O, characterized by elevated levels of CD4^+^ lymphocytes rather than CD8^+^ lymphocytes. Moreover, basal *Il17* messenger RNA (mRNA) levels were lower while *Il22* mRNA levels were higher in HFD-O, and we observed the same pattern after LPS challenge.

**Discussion:**

Further examination of myeloid cells isolated from bone marrow and allowed to differentiate showed that HFD-O macrophages displayed an anti-inflammatory phenotype. Additionally, treatment with the m1mAChR agonist contributed to reducing inflammatory marker levels in both groups. In summary, our findings demonstrate that HFD-O are protected against LPS-induced sepsis, and this protection is mediated by the central m1mAChR. Moreover, the inflammatory response in the liver, spleen, and bone marrow-differentiated macrophages is diminished. However, more extensive analysis is necessary to elucidate the specific mechanisms by which m1mAChR modulates the immune response during sepsis.

## 1 Introduction

Obesity paradoxically exhibits an association with improved mortality outcomes in sepsis when compared with leaner patients (1, 2). This phenomenon, known as the obesity paradox, has been discussed previously (3, 4). However, it is important to note that the protective effect of obesity in sepsis remains a topic of debate (5). Some studies have demonstrated the beneficial impact of obesity on sepsis outcomes (6, 7). Conversely, other studies have found that after adjusting for comorbidities, the effect of obesity on sepsis outcomes becomes statistically insignificant (8–10). Furthermore, the precise mechanisms underlying the protective function of overweight and obesity in sepsis remain poorly understood, although some studies have proposed that energy stores in adipose tissue and differential inflammatory responses in individuals with obesity may play an important role (3, 11, 12).

The central nervous system plays a critical role in communicating with the immune system, with the vagus nerve being particularly important (13). The regulation of inflammatory responses mediated by the vagus nerve is referred to as the cholinergic anti-inflammatory pathway (CAP) with participation of cholinergic receptors, the α7 nicotinic acetylcholine receptor (α7nAChR) and the m1 muscarinic acetylcholine receptor (m1mAChR) (14–16).

Stimulation of the CAP can attenuate inflammatory responses in sepsis (17). The JAK2/STAT3 pathway plays a crucial role in the anti-inflammatory effects associated with α7nAChR activation (18, 19), downregulating NF-κB binding to DNA, subsequently reducing cytokine expression (20). In a previous study from our research group, we observed that short-term consumption of a high-fat diet (HFD) for 3 days resulted in reduced expression of hypothalamic α7nAChR and increased mortality in C57/BL6 mice following sepsis induced by administration of lipopolysaccharide (LPS) or caecal ligation and puncture (CLP). Moreover, HFD consumption impaired the ability of PNU (a specific agonist of α7nAChR) to reduce inflammatory markers after LPS injection, thereby contributing to a higher probability of death in sepsis (21).

The global increase in obesity has contributed to a rise in pre-pregnant and maternal obesity, which has long-term implications for the health of both mothers and their offspring (22–24). Animal studies utilizing rodent and non-human primate models have demonstrated that maternal obesity induced through dietary interventions leads to various health issues in the offspring, including obesity, diabetes, hypertension, fatty liver, and behavioural changes (25–29). However, there are few studies available regarding the impact of maternal obesity on the inflammatory response in offspring. Studies conducted on both rodent models and humans have uncovered that maternal obesity can instigate significant modifications in the immune response, microbiota, and the development of the immune system (30–33).

Therefore, our objective was to investigate the impact of maternal HFD consumption on the systemic inflammatory response and sepsis susceptibility in the offspring.

## 2 Materials and methods

### 2.1 Animals

**Figure 1:**
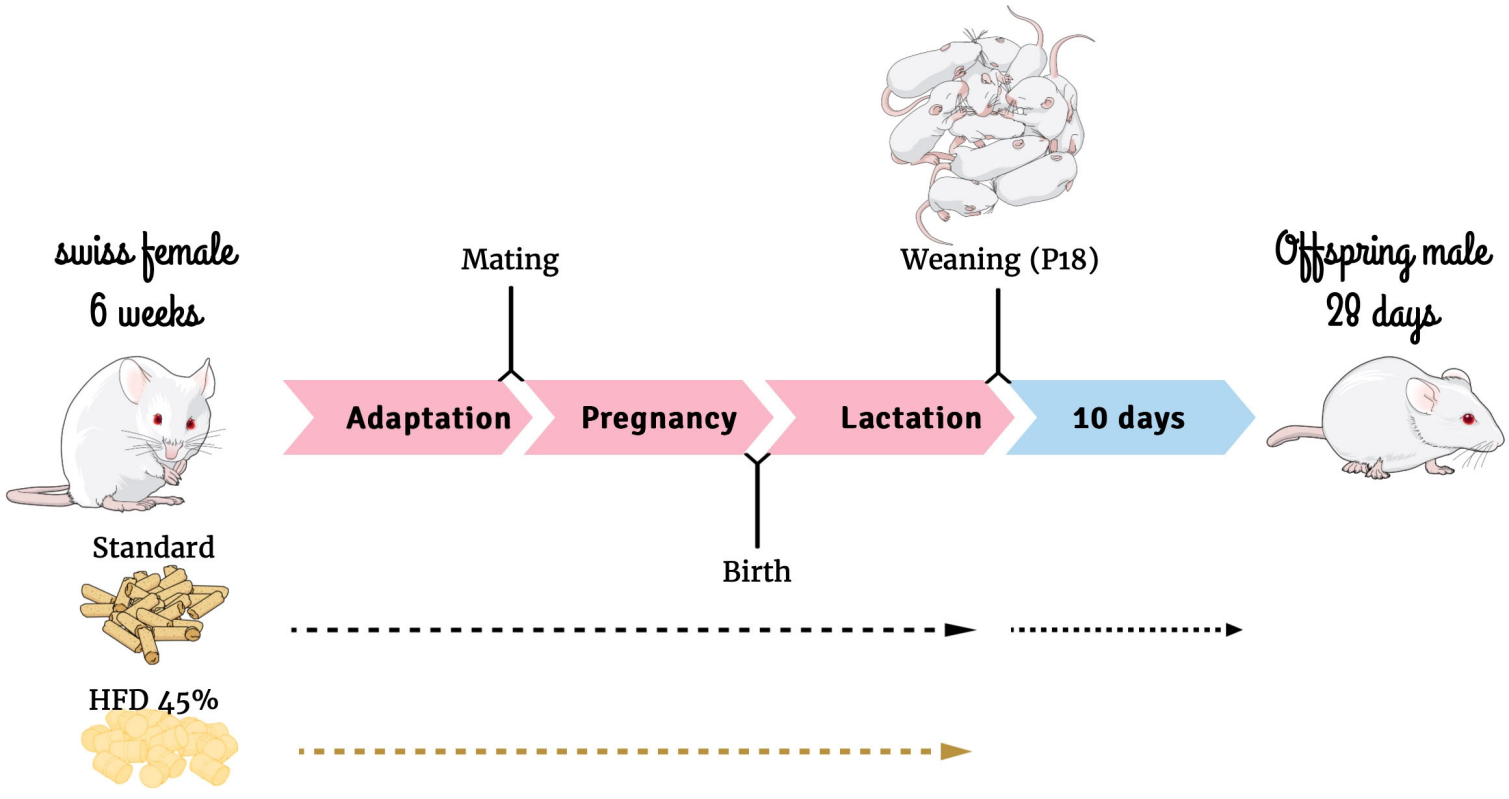
Experimental design. Mind the Graph website was used to prepare **Figure 1**.

Five-week-old Swiss female mice were obtained from the Multidisciplinary Center for Biological Research at the University of Campinas (Campinas, Brazil). The mice were kept in a temperature-controlled environment with a 12-h photoperiod. Experiments were performed in accordance with the ethical guidelines and regulations for use of laboratory animals. Ethics approval for this study, including the design for sepsis development and mortality analysis presented in this paper, was obtained from the State University of Campinas Ethics Committee (Protocol 5733-1). Importantly animals did not experience any form of suffering throughout the study. The female mice were randomly separated into two groups (25 dams per group), fed with either a HFD or a standard diet (SC) (NUVILAB^®^ Cr-1, Nuvital, PR, Brazil) (**Table 1**) for 4 weeks *ad libitum* before mating. Dams continued with the diet during pregnancy and lactation. After birth, the litter size was culled to eight mice per litter. Male offspring were weaned on postnatal day 18 (P18) and fed with standard chow until P28 (**Figure 1**). Each experimental protocol, such as surgery and survival tests, was conducted using one pup from each dam to constitute the respective group. Offspring at different ages were utilized for the receptor expression experiment, specifically at birth (P0), P56, and P82. The HFD was prepared in our laboratory according to the AIN-93G but modified for high-fat content (45%) as described previously (34).

**Table 1:** Primers sequence for quantitative polymerase chain reaction.

Gene	Forward sequence (5′→3′)	Reverse sequence (5′→3′)
*Il17*	AACCGTTCCAC GTCACCCT	GCACTGAGCTTCCCAGATCAC
*Il22*	CGGCTCATCGG GGAGAAAC	TGACTGGGGGAGCAGAACG
*Tgfb*	CAACCCAGGTCC TTCCTAAA	GGAGAGCCCTGGATACCAAC
*Ifng*	TGAGCTCATTGA ATGCTTGG	ACAGCAAGGCGAAAAAGGAT
*Nos2*	GCCACCAACAAT GGCAACA	CGTACCGGATGAGCTGTGAATT
*Arg1*	AACACGGCAGTG GCTTTAACC	GGTTTTCATGTGGCGCATTC

### 2.2 Anaesthesia and tissue extraction

Mice were anaesthetized with a mixture containing ketamine (139.2 mg kg^-1^ body weight [bw]), diazepam (4 mg kg^-1^ bw), and xylazine (18.4 mg kg^-1^bw) and subsequently euthanized by decapitation for tissue collection. Tissue samples were frozen in liquid nitrogen and stored at -80°C until processing. Isoflurane, an inhalational anaesthetic, was used for induction and maintenance of general anaesthesia during stereotaxic surgery. It was used at 3%–4% for induction and reduced to 2% during surgery.

### 2.3 Inflammatory response

The offspring were separated as described below to evaluate the inflammatory response.

Design 1

An LPS-induced sepsis mouse model was used. Mice were treated with a lethal dose of LPS diluted in sterile saline and administered intraperitoneally (IP) at 30 mg kg^-1^ bw. The offspring were observed for 72 h. The survival rate was recorded every 1 h. In the survival study, mice were allowed *ad libitum* access to food and water. The experiment was replicated twice to validate the obtained results.

In a separate study, mice were treated intracerebroventricularly (ICV) with benztropine (mesylate) 20 min before the LPS challenge. Benztropine, an m1mAChR antagonist, or phosphate-buffered saline (PBS) was injected at 40 µg kg^-1^ bw. The mice were sacrificed 10 h after LPS administration; the serum was collected for analysis. The time was defined based on the survival rate.

Design 2

To explore the underlying mechanisms of central m1mAChR-mediated anti-inflammatory effects in mice, McN-A-343, an m1mAChR agonist, was administered ICV at 5 ng kg^-1^ bw. The mice were euthanized 2 h after injection. In a separate study, the mice were treated with an agonist (ICV) 20 min before LPS challenge (1 mg kg^-1^ bw IP). The mice were euthanized 2 h after LPS challenge.

### 2.4 Immunofluorescence analysis

At P28, offspring of both groups (SC-O and HFD-O) were perfused with 4% paraformaldehyde (PFA). Then, the brains were extracted and fixed in 4% PFA. Subsequently, the brains were embedded in Tissue-Tek (Sakura, Torrance, CA, USA), frozen, and cut into 15-µm thick coronal sections, following *The Rat Brain in Stereotaxic Coordinates* by Charles Watson and George Paxinos. The slides were incubated in blocking solution (3% bovine albumin; Sigma-Aldrich, St. Louis, MO, USA) for 90 min, followed by incubation with specific primary antibodies overnight at 4°C. The primary antibodies used were anti-Chrm1 diluted 1:500 (sc-365966, Santa Cruz Biotechnology, Inc, California) and anti-F480 diluted 1:500 (ab6640, Abcam, Inc, Boston). The slides were washed and incubated with appropriate secondary antibodies for 120 min. The secondary antibodies used were Donkey anti-rabbit conjugated to Alexa 488 diluted 1:500 (A-21206, Thermo Fisher Scientific, Inc, Waltham, MA) and goat anti-Rat conjugated to Cy3 diluted 1:1000 (ab6953, Abcam). TO-PRO-3 Iodide was used for nuclear labelling (1:1000; Life Technologies Inc, Carlsbad, CA). The slides were visualized and images were captured using a TCS SP5II Leica confocal microscope (Leica Microsystems, Wetzlar, Germany).

### 2.5 Serum measurements

The offspring were sacrificed by decapitation on P28 after overnight fasting, and blood was collected. The samples were centrifuged at 1300 rpm for 15 min at room temperature. The serum was collected and stored at -80°C until processing. The cytokine levels were measured using DuoSet enzyme-linked immunosorbent assay kits, DY410-05 mouse TNF; DY401-05 mouse IL1-β/IL1-F2; and DY417-05 mouse IL-10 (R&D Systems, Minneapolis, MN, USA). CD14 levels were measured using Mouse CD14 Quantikine ELISA Kit, #MC140 (R&D Systems, Minneapolis, MN, USA. The C-reactive protein (CRP- K059-8.1) and albumin levels (K040-1) were obtained from biochemical analysis provided by BIOCLIN (Quimica Basica LTDA, Belo Horizonte, Brazil).

### 2.6 Stereotaxic surgery

The offspring from control and HFD dams received 3%–4% isoflurane inhalational anaesthesia and were placed in a stereotaxic instrument (Stoelting Co. Wood Dale, Illinois). Isoflurane was reduced to 2% during surgery for cannula implantation. Afterwards, a 26G needle was used for the cannula and inserted into the lateral ventricle through a cranial incision. The following coordinates relative to the bregma were used to access the lateral ventricle: anterior/posterior axis, 0.34 mm from bregma to the rear; lateral, 1 mm from the midline; dorsoventral, 2.2 mm from the surface of the skull. Dental acrylic glue was added to secure the cannula following correct positioning. After surgery, the animals were allowed to recover from anaesthesia on a warm pad. Carprofen, an analgesic, was administered for postoperative pain (5 mg kg^-1^ bw, IP). The cannula placement was tested 6 days after the surgery by measuring the dipsogenic response to angiotensin II injection (2 µL of a 1 × 10^-6^M solution, ICV) (Sigma-Aldrich Inc, MERK, St Louis, MO). The time and dose of McN-A-343 (agonist) and benztropine (antagonist) treatment were standardized from time-course and dose-response experiments (data not shown).

### 2.7 Isolation of bone marrow cells

Bone marrow cells were isolated as described previously (21). The long bones (femur and tibia) were removed from the offspring and placed in 0.5 mL perforated tubes inside 1.5 mL tubes, which were then centrifuged at 1200 g for 15 s at 4°C. The cell pellet was resuspended in Roswell Park Memorial Institute (RPMI) 1640 culture medium (Invitrogen, Carlsbad, CA, USA) supplemented with 10% foetal bovine serum (FBS; Invitrogen) and 1% penicillin (100 U/mL)/streptomycin (100 μg/mL) (Invitrogen). The cells were counted with a Neubauer chamber and placed on 60 mm culture dishes. The cells were cultivated for 7 days at 37°C in an atmosphere containing 5% CO_2_ and 95% humidity. After this period, photos were taken of the culture, and cells were trypsinized and collected for western blotting, RT-PCR, and flow cytometry analysis.

### 2.8 Western blotting analysis

Tissues were homogenized in freshly prepared ice-cold buffer (1% v/v Triton X-100, 0.1 M Tris, pH 7.4, 0.1 M sodium pyrophosphate, 0.1 M sodium fluoride, 0.01 M EDTA, 0.01 M sodium vanadate, 0.002 M PMSF (Phenylmethylsufonyl fluoride), and 0.01 mg mL^-1^ aprotinin). The samples were centrifuged at 12,000 rpm for 30 min at 4°C. The supernatant was removed and the protein concentration was determined using the Bradford dye-bleeding method. The samples were resuspended in Laemmli sample buffer and boiled for 5 min before separation by SDS-PAGE using a miniature slab gel apparatus (Bio-Rad, Richmond, CA, USA). The separated proteins were electrotransferred from the gel to a nitrocellulose membrane for 30 min in a transfer buffer that contained methanol and SDS. These membranes were incubated overnight at 4°C with specific antibodies: α7nAChR (bs-1049R, Bioss Antibodies Inc, Woburn, MA), phosphorylated JNK (#9255; Cell Signaling Technology Inc, Danvers, MA), phosphorylated STAT3 (#9145, Cell Signaling), GAPDH (sc-32233, Santa Cruz Biotechnology, Inc., California), and phosphorylated NF-κB (#30335, Cell Signaling Technology Inc, Danvers, MA). Then, after washing with Tris-buffered saline (TBS)-Tween 20 (TTBS; 10 mM Tris, 150 mM NaCl, 0.5% Tween 20), the nitrocellulose membranes were probed with peroxidase-conjugated secondary antibodies (KPL, Gaithersburg, MD, USA) for 90 min at room temperature. Proteins were detected by a chemiluminescence kit (SuperSignal West Pico Chemiluminescent Substrate, Thermo Fisher Scientific Inc) and bands were evaluated by densitometry using Scion Image software (ScionCorp, MD, USA). The intensities of the bands were normalized to the loading control (GAPDH).

### 2.9 RT-PCR analysis

Frozen tissues were homogenized in TRIzol reagent (Life Technologies) for RNA extraction according to the manufacturer’s instructions. After incubation for 5 min at room temperature for complete dissociation, chloroform was added to the homogenate. Following centrifugation, the RNA phase was precipitated with isopropyl alcohol and the pellet was washed with 75% and 100% ethanol. After drying, the pellet was resuspended in ultra-pure water and stored at -80°C. RNA was quantified with a Nanodrop ND-2000 (Thermo Fisher Scientific). Reverse transcription was performed with 3 µg of total RNA using the High-Capacity cDNA Reverse Transcription kit (Life Technologies). Relative expression was determined using TaqMan Gene Expression Assays (Thermo Fisher Scientific) and SYBR Green Master Mix (Bio-Rad). The following TaqMan Gene Expression Assays were used: *Chrna7* (Mm01312230_m1), *Il6* (Mm01312230_m1), *Tnf* (Mm00443258_m1), *Il1b* (Mm00434228_m1), *Socs3* (Mm00545913_s1), and *Il10* (Mm01288386_m1). *Gapdh* (4351309; Applied Biosystems, USA) was used as endogenous control.

Quantitative PCR was performed with the SYBR Green Master Mix (Bio-Rad). The primers used are listed in **Table 1**. Real-time PCR was performed on an AB/Prism 7500 fast platform. The data were analysed using the Sequence Detection System 2.0.5 software.

### 2.10 Flow cytometry analysis

Cells isolated from the spleen and bone marrow were submitted to flow cytometry analysis. The spleen and bone marrow cells were evaluated with a macrophage panel, which determined the number of CD45^+^, F480^+^, CD11c^+^, and CD206^+^ cells. Spleen cells were also evaluated with a lymphocyte panel, which determined the number of CD3^+^, CD4^+^, CD8^+^, and Ly6 G^+^ cells.

Spleens were collected, and then gently dissociated by a needle and rinsed with PBS. Single-cell suspensions (1 × 10^6^) were then suspended in DMEM/FBS). The cells were treated with specific antibodies conjugated to FITC (CD45; CD206), PECy7 (CD45), APC (F480; CD4), PE (CD11c; Ly6G; CD8), and Alexa 488 (CD3). The incubation was at room temperature for 15 min protected from light. The analysis was performed in BD-FACS Accuri Cytometry (Becton Dickinson, MD, USA) and 10,000 events were acquired. The data were analysed using the FlowJo 7.6 software.

Bone marrow cells were collected after 7 days of spontaneous differentiation. The cells were treated with specific antibodies conjugated to FITC (CD206), PECy7 (CD45), APC (F480), and PE (CD11c). The incubation was at 4°C for 15 min protected from light. Analysis was performed in BD-FACS Accuri Cytometry (Becton Dickinson) and 10,000 events were acquired. The data were analysed using the FlowJo 7.6 software.

### 2.11 Data presentation and statistical analysis

The results are presented as the mean ± standard error. The data were evaluated with the Kolmogorov–Smirnov test to determine whether they were normally distributed. After confirming a normal distribution, Student’s t-test for unpaired samples or analysis of variance (ANOVA) was used. ANOVA was followed by the Bonferroni *post hoc* test to determine differences between more than two groups. The log-rank test was used to analyse the survival rate. Statistical significance for all analyses was set at p < 0.05. All statistical comparisons were performed using GraphPad Prism 9.5.3 (GraphPad Software, San Diego, CA, USA).

## 3 Results

### 3.1 Maternal HFD consumption protects HFD-O against sepsis mortality

In the study, we administered a lethal dose of LPS to SC-O and HFD-O to induce sepsis and then recorded their survival rates. First, we assessed whether the LPS challenge was effective in inducing sepsis in both groups. We measured biomarkers of sepsis and the inflammatory response in the serum of the offspring 10 h after the LPS challenge.

The CRP levels were higher after LPS treatment, with no significant difference between SC-O and HFD-O. Albumin levels did not show any significant differences between the groups. CD14 levels were elevated in all offspring treated with LPS and exceeded the highest point of the standard curve (**Table 2**). TNF, IL1-β, and IL-10 were undetectable in SC-O and HFD-O that were not challenged with LPS. TNF and IL-10 levels were lower in HFD-O compared with SC-O after LPS injection. However, this decrease was prevented by treatment with an m1mAChR antagonist. IL1-β was only detected in SC-O after LPS injection and in HFD-O treated with the m1mAChR antagonist (**Table 2**). We also evaluated the spleen weight. LPS significantly increased the spleen weight in SC-O compared with SC-O that were not treated with LPS (**Table 2**). However, there was no difference in spleen weight in HFD-O treated with LPS.

**Table 2:** Serum biomarkers of sepsis in the offspring.

		Control offspring (SC-O)		Hight-fat diet offspring (HFD-O)	
	Basal	LPS	Basal		LPS
**Benztropine (antagonist)**	**--**	**--**	**--**	**--**	**+**
CPR (mg/L)	2.718 ± 0.0412	3.155 ± 0.0658*	2.794 ± 0.032	3.052 ± 0.054*	3.018 ± 0.111*
Albumin (g/dL)	3.168 ± 0.131	3.168 ± 0.131	2.882 ± 0.155	2.944 ± 0.056	3.008 ± 0.089
CD14 (pg/mL)	7.953 ± 0.004	Overage	Overage	Overage	Overage
TNF (pg/mL)	Not detected	202.48 ± 21.34	Not detected	97.89 ± 27.39*	201.23 ± 33.68#
IL-1β (pg/mL)	Not detected	469.63 ± 82.66	Not detected	Not detected	81.15 ± 16.97
IL-10 (pg/mL)	Not detected	93.39 ± 25.54	Not detected	39.46 ± 10.63	137.39 ± 33.90#
Spleen weight (g/100 g body weight)	0.448 ± 0.023	0.544 ± 0.045*	0.431 ± 0.020	0.493 ± 0.026	0.597 ± 0.060

The data represent the mean ± standard deviation (n = 6 per group).

The data were analysed with analysis of variance.

*Significant difference (p < 0.05) between basal and lipopolysaccharide (LPS) treatment.

#Significant difference (p < 0.05) difference between HFD-O and SC-O.

**Figure 2:**
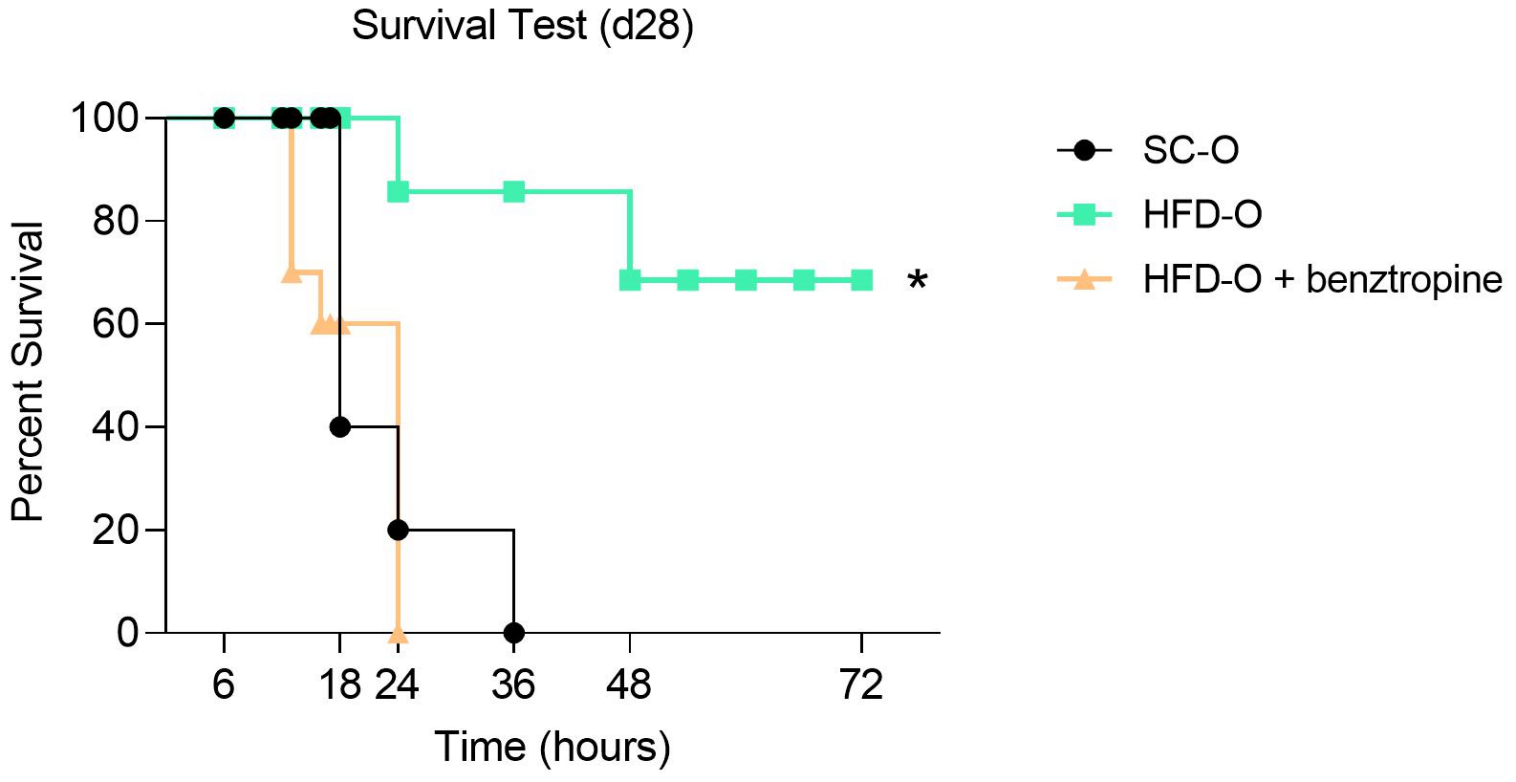
Survival analysis of the offspring. Sepsis was induced with a lethal dose of lipopolysaccharide (LPS; 30 mg LPS kg^-1^ body weight, intraperitoneal) administered to control offspring (SC-O, n = 10) and high-fat diet offspring (HFD-O, n = 10) at postnatal day 28. Some HFD-O were pretreated for 20 min with the m1mAChR antagonist benztropine (intracerebroventricular) (HFD-O + Benztropine, n = 10). The mice were observed for 72 h, and the survival rate was recorded every 1 h. The data represent the mean ± standard error of the mean. *p < 0.05 HFD-O versus SC-O and HFD-O + Benztropine versus HFD-O (log-rank test).

Considering that LPS was sufficient to activate the inflammatory response, we assessed the SC-O and HFD-O survival curves. The mortality rate was significantly higher in SC-O compared with HFD-O (**Figure 2**). However, prior ICV treatment with benztropine (mesylate), a pharmacological antagonist of m1mAChR, increased the mortality of HFD-O to levels comparable to SC-O (**Figure 2**). This finding suggests that central m1mAChR is involved in protecting HFD-O against sepsis.

### 3.2 Maternal HFD consumption leads to higher central m1mAChR expression in HFD-O

**Figure 3:**
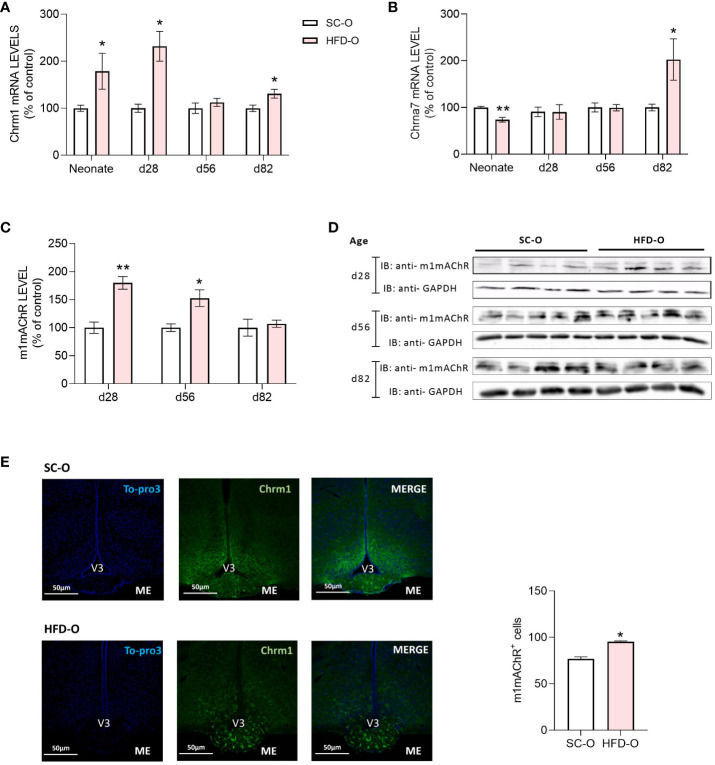
Cholinergic anti-inflammatory pathway receptor expression in the hypothalamus of the offspring. Hypothalamic m1mAChR and α7nAChR messenger RNA (mRNA) **(A, B)** and protein **(C, D)** levels were evaluated by RT-PCR and western blot, respectively, in control and high-fat diet offspring (SC-O and HFD-O, respectively). The mRNA and protein levels were evaluated at birth (neonate) and postnatal days 28, 56, and 82. Molecular weight of proteins: m1mAChR – 52KDa; α7nAChR – 55KDa; and GAPDH – 35KDa. The percent expression of control (GAPDH) is shown (mean ± standard error of the mean, n = 5 pups per group). Asterisks indicate significant differences determined by Student’s t-test (*p < 0.05 and **p < 0.01). Confocal images illustrating m1mAChR^+^ cells (green) and nuclear labelling with TO-PRO-3 (blue) in coronal brain sections (15 µm thick) from 28-day-old offspring **(E)**. Scale of images 50 µm. The number of m1mAChR^+^ cells in the median eminence (ME) of SD-O and HFD-O (n = 3 per group) **(E)**. V3: third ventricle.

We evaluated the distribution and expression of cholinergic receptors (α7nAChR and m1mAChR) in the hypothalamus (**Figure 3**). First, we evaluated the mRNA levels of both receptors at P0, P28, P56, and P82. While m1mAChR mRNA expression was higher in HFD-O at P0, P28, and P82 compared with SC-O (**Figure 3A**), α7nAChR mRNA expression in HFD-O was reduced at P0 and increased at P82 compared with SC-O (**Figure 3B**). Additionally, we evaluated m1mAChR protein expression by western blot at P28, P56, and P82. We noted increased hypothalamic m1mAChR protein expression in HFD-O at P28 and P56 compared with SC-O (**Figures 3C, D**). Considering the higher hypothalamic expression of m1mAChR, we evaluated he distribution of m1mAChR expression with immunofluorescence at P28 (**Figure 3E**). There were more m1mAChR^+^ cells in the median eminence of HFD-O compared with SC-O (**Figure 3E**). Nevertheless, m1mAChR expression seems to be higher in the arcuate nucleus compared with the median eminence of SC-O (**Figure 3E**).

### 3.3 m1mAChR reduces inflammatory pathway activation in the liver of HFD-O

**Figure 4:**
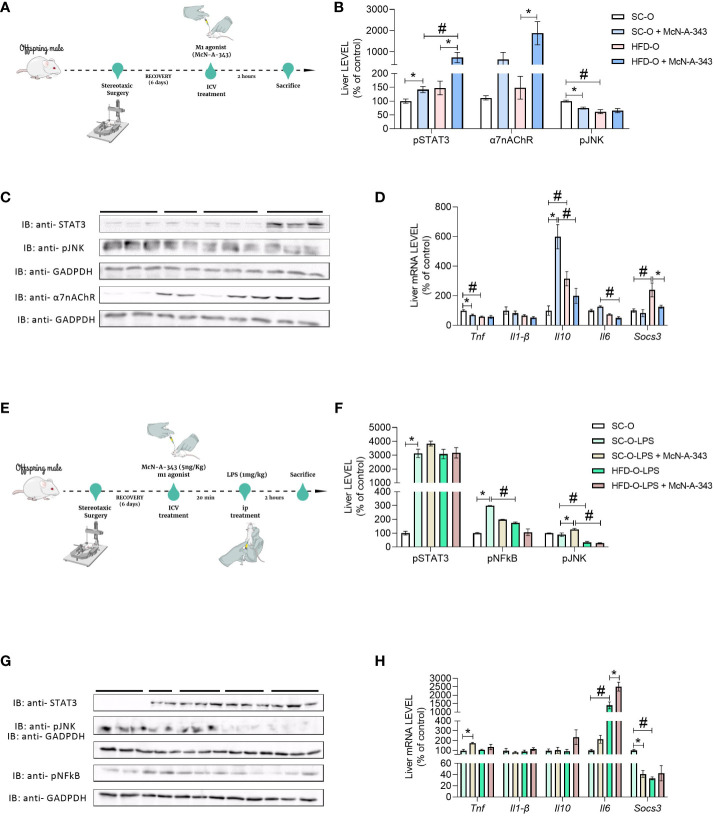
Inflammatory markers in the liver of the offspring. Experimental design for pharmacological treatment with the m1mAChR agonist McN-A-343 (delivered intracerebroventricularly [ICV]) **(A)**. Hepatic pSTAT3, α7nAChR, and pJNK protein expression **(B, C)** and *Tnf*, *Il1b*, *Il10*, *Il6*, and *Socs3* messenger RNA (mRNA) expression **(D)** were evaluated by western blot and RT-PCR, respectively, in 28-day-old standard and high-fat diet offspring (SC-O and HFD-O, respectively). The mice were treated with the m1mAChR agonist McN-A-343 (5 ng kg^-1^, ICV). Experimental design for simultaneous administration of lipopolysaccharide (LPS; 1 mg kg^-1^, intraperitoneal) and the m1mAChR agonist (ICV) **(E)**. Hepatic pSTAT3, pNF-κB, and pJNK protein expression **(F, G)** and *Tnf*, *Il1b*, *Il10*, *Il6*, and *Socs3* mRNA expression **(H)** were evaluated by western blot and RT-PCR in 28-day-old SC-O and HFD-O. Molecular weight of proteins: pSTAT3 – 90KDa; α7nAChR – 55KDa; pJNK – 55KDa; pNFkB – 65KDa; and GAPDH – 35KDa. The data represent the mean ± standard error of the mean (n = 5 per group). The data were analysed with analysis of variance. *Significant difference (p < 0.05) between basal and agonist or LPS treatment. #Significant difference (p < 0.05) between HFD-O and SC-O. Mind the Graph website was used to prepare **Figures 4A, E**.

To assess the impact of central m1mAChR activation on liver signalling pathways and α7nAChR expression in offspring at P28, we administered McN-A-343 (ICV), a pharmacological agonist of m1mAChR (**Figure 4A**). **Figures 4B, C** indicate that hypothalamic activation of m1mAChR increased α7nAChR and phosphorylated STAT3 (pSTAT3) protein expression in the liver of SC-O and HFD-O. Notably, pSTAT3 expression was higher in the liver of HFD-O than SC-O. Additionally, m1mAChR activation was accompanied by a reduction in phosphorylated JNK (pJNK) expression in the liver (**Figures 4B, C**).

We also evaluated liver cytokine mRNA levels. *Il1b* expression was not different between the groups, indicating similar levels of this cytokine. Interestingly, *Tnf* expression in HFD-O liver was lower compared with SC-O liver. However, treatment with the m1mAChR agonist (McN-A-343) via ICV administration reduced *Tnf* mRNA expression in SC-O liver, but there was no additional effect in HFD-O liver (**Figure 4D**). *Il10* mRNA expression increased in HFD-O liver compared with SC-O liver, but ICV administration of McN-A-343 significantly increased *Il10* mRNA expression in SC-O liver compared with HFD-O liver. Furthermore, McN-A-343 delivered via ICV administration demonstrated a greater inhibitory effect on liver *Il6* mRNA expression in HFD-O compared with SC-O. Similarly, the mRNA levels of *Socs3*, an important regulatory molecule in inflammation, were higher in HFD-O liver (**Figure 4D**). This indicates that maternal HFD consumption may lead to increased SOCS3 expression in HFD-O liver, potentially impacting the regulation of inflammatory responses.

As shown in the **Figure 4E**, we investigated the hepatic inflammatory response in SC-O and HFD-O after LPS treatment. We simultaneously administered SC-O and HFD-O LPS (1 mg kg^-1^, IP) and an m1mAChR agonist. Liver pSTAT3, a component of the anti-inflammatory pathway, was increased in all LPS-challenged mice. This suggests activation of the anti-inflammatory response in both SC-O and HFD-O following LPS treatment. Moreover, phosphorylated NF-κB (pNF-κB) and pJNK expression was decreased in the liver of LPS-treated HFD-O compared with LPS-treated SC-O. This indicates that maternal HFD consumption may have modulated the hepatic inflammatory response in the offspring, resulting in reduced activation of these pro-inflammatory signalling pathways. Interestingly, when we administered LPS-treated HFD-O with an m1mAChR agonist, there was a further decrease in pNF-κB and pJNK expression in the liver (**Figures 4F, G**). This suggests that activation of m1mAChR can enhance the anti-inflammatory response and attenuate the activation of pro-inflammatory signaling pathways in the liver of HFD-O following LPS challenge, as depicted in **Figure 4H**. These findings indicate that maternal HFD consumption and m1mAChR activation can influence the hepatic inflammatory response in offspring, potentially leading to a more pronounced anti-inflammatory state and reduced activation of pro-inflammatory pathways in HFD-O following LPS treatment.

### 3.4 m1mAChR activates the lymphocyte response in the spleen of HFD-O

The inflammatory and immune response to pathogens depends on the spleen. We investigated the levels of inflammatory cytokines in the spleen of SC-O and HFD-O following ICV treatment with the m1mAChR agonist. However, there were no significant differences in the inflammatory markers between the groups (data not shown).

**Figure 5:**
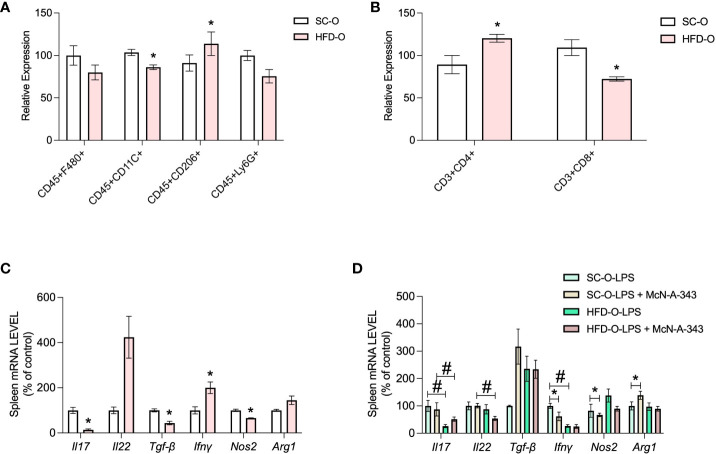
Immune response markers in the spleen of the offspring. Splenic CD45^+^F480^+^, CD45^+^CD11c^+^, CD45^+^CD206^+^, and CD45^+^Ly6G^+^ cells were evaluated with the macrophage panel **(A)** and CD3^+^CD4^+^ and CD3^+^ CD8^+^ cells were evaluated with the lymphocyte panel **(B)** by flow cytometry. The data represent expression of high-fat diet offspring (HFD-O) relative to the control offspring (SC-O). Splenic *Il17*, *Il22*, *Tgfb*, *Ifng*, *Nos2*, and *Arg1* messenger RNA (mRNA) expression **(C)** was evaluated by RT-PCR in 28-day-old SC-O and HFD-O. Splenic *Il17*, *Il22*, *Tgfb*, *Ifng*, *Nos2*, and *Arg1* mRNA expression **(D)** was evaluated in the offspring following lipopolysaccharide (LPS) challenge (1 mg kg^-1^, intraperitoneal) and treatment with the m1mAChR agonist McN-A-343 (5 ng kg^-1^, intracerebroventricular). The data represent the mean ± standard error of the mean. The data were analysed with analysis of variance. *Significant difference (p < 0.05) between basal and agonist or LPS treatment. #Significant difference (p < 0.05) between HFD-O and SC-O.

Next, we performed flow cytometry using macrophage and lymphocyte panels to evaluate the inflammatory response of the spleen. In the macrophage panel, there was a decrease in CD45^+^CD11c^+^ cells in HFD-O compared with SC-O, and an increase in CD45^+^CD206^+^ cells in HFD-O (**Figure 5A**). These findings were supported by the differential expression of macrophage markers in the spleen. Similarly, *Nos2* mRNA expression was decreased in HFD-O compared with SC-O (**Figure 5C**), whereas *Arg1* mRNA expression appeared to be increased in HFD-O (**Figure 5C**). Furthermore, treatment with the m1mAChR agonist decreased *Nos2* mRNA expression and increased *Arg1* expression only in SC-O after LPS challenge (**Figure 5D**). Taken together, these observations confirm that the macrophages present in the spleen exhibit an anti-inflammatory profile.

The lymphocyte panel revealed an increase in CD3^+^CD4^+^ cells in the spleen of HFD-O compared with SC-O, and a reduction in CD3^+^CD8^+^ cells in HFD-O (**Figure 5B**). These findings were accompanied by a decrease in *Il17* and *Tgfb* mRNA expression in the spleen of HFD-O compared with SC-O (**Figure 5C**). To investigate the increase in T-helper lymphocytes, we determined the specific type of T-helper lymphocytes present in the spleen of HFD-O after LPS challenge and treatment with an m1mAChR agonist. Interestingly, we observed a reduction in *Il17* mRNA expression in the presence of LPS (**Figure 5D**), while *Il22* mRNA expression appeared to be increased in HFD-O (**Figure 5C**). These observations highlight the presence of distinct lymphocyte profiles in HFD-O. Additionally, *Ifng* mRNA expression was decreased in HFD-O following LPS challenge, and the m1mAChR agonist reduced *Ifng* mRNA expression in LPS-treated SC-O (**Figure 5D**). Overall, these findings suggest reduced differentiation of lymphocytes towards the Th17 profile in HFD-O in the basal state and after LPS challenge.

### 3.5 m1mAchR activates bone marrow cell differentiation in anti-inflammatory macrophages profile in HFD-O

**Figure 6:**
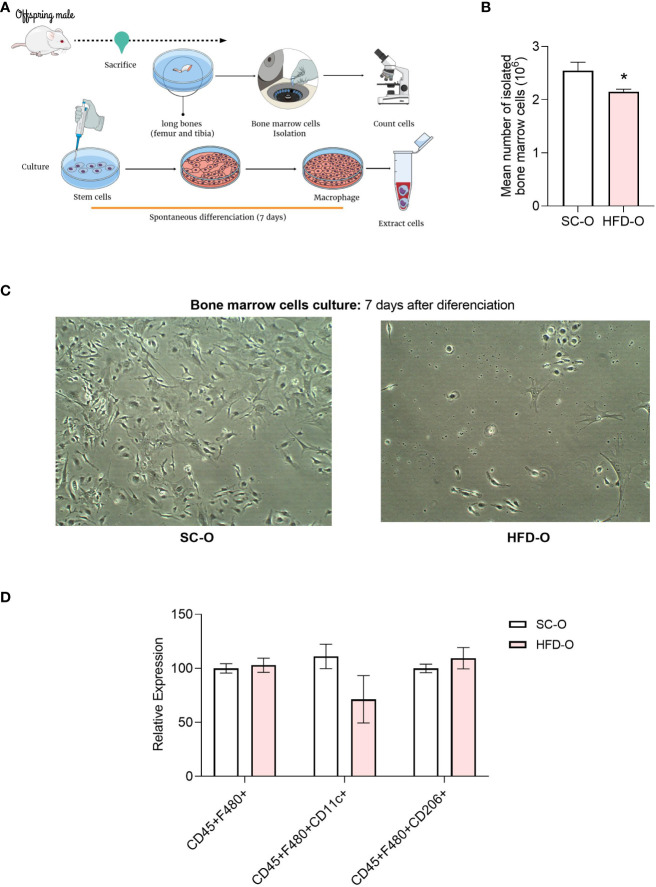
Profile of bone marrow cells after differentiation. Bone marrow cells were isolated from the long bones of 28-day-old control and high-fat diet offspring (SC-O and HFD-O, respectively) **(A)**. The cells were counted before the culture **(B)**. After 7 days of spontaneous differentiation, pictures were taken of the culture **(C)** to confirm that the cells had differentiated into macrophages. CD45^+^F480^+^, CD45^+^CD11c^+^, and CD45^+^CD206^+^ cells were evaluated with the macrophage panel by flow cytometry **(D)**. The data represent the mean ± standard error of the mean. *Significant difference (p < 0.05) based on Student’s t-test. Mind the Graph website was used to prepare **Figure 6A**.

To investigate the relationship between central muscarinic receptor and macrophage activation, we isolated bone marrow cells from the offspring. We cultured these cells and after 7 days of differentiation, we submitted them for further analysis. We counted the number of cells with a Neubauer chamber immediately after isolation (**Figure 6A**). There were fewer cells isolated from HFD-O compared with SC-O (**Figure 6B**). Additionally, a representative image shows that after 7 days of differentiation, the number of macrophages appears to be reduced in HFD-O compared with SC-O (**Figure 6C**). However, when evaluating the macrophage panel with flow cytometry, there were no significant differences between the groups in terms of the number of positive cells for either inflammatory or anti-inflammatory markers (**Figure 6D**).

**Figure 7:**
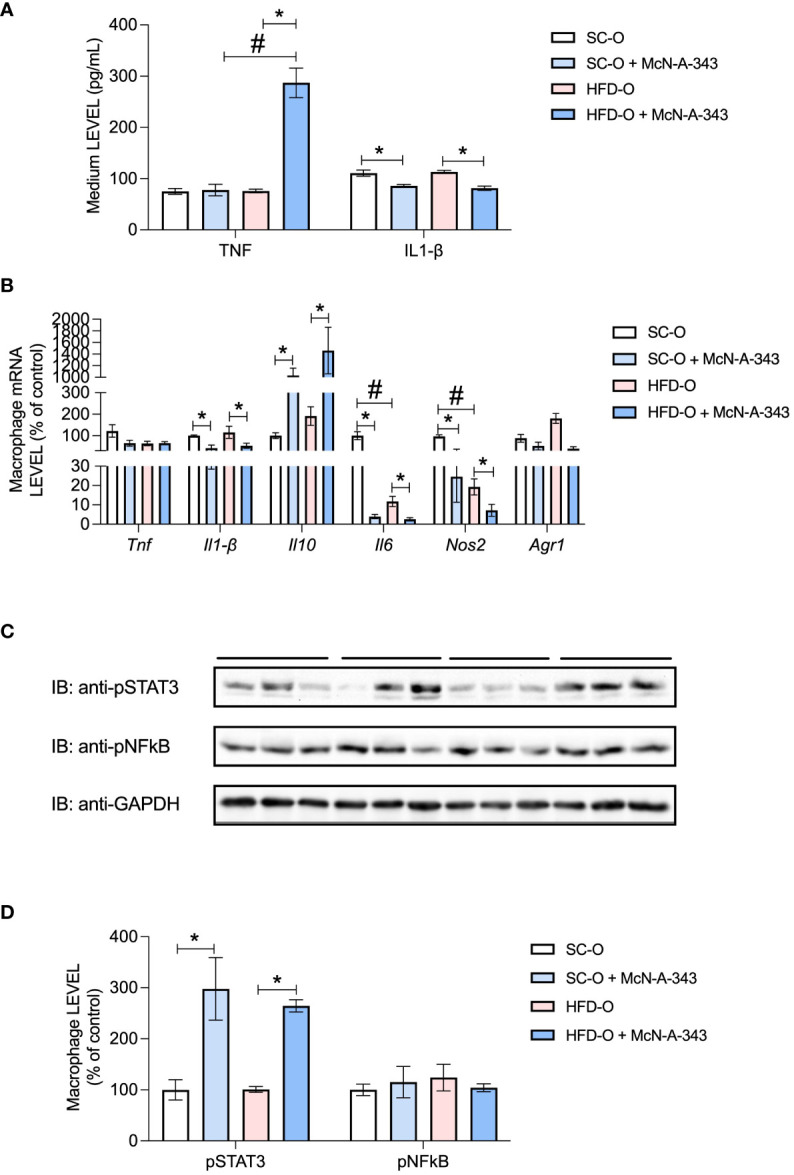
Inflammatory markers in bone marrow cells after differentiation. TNF and IL-1β protein expression was evaluated with enzyme-linked immunosorbent assays **(A)** in the culture medium after 7 days of spontaneous differentiation. *Tnf*, *Il1b*, *Il10*, *Il6*, *Nos2*, and *Arg1* messenger RNA (mRNA) expression **(B)** and pSTAT3 and pNF-κB protein expression **(C, D)** were evaluated by RT-PCR and western blot, respectively, in macrophages differentiated from control and high-fat diet offspring (SC-O and HFD-O, respectively) bone marrow. The mice were treated with the m1mAChR agonist McN-A-343 (5 ng kg^-1^, intracerebroventricular). The bars at the top of blots represent the groups. The data represent the mean ± standard error of the mean. Molecular weight of proteins: pSTAT3 – 90KDa; pNFkB – 65KDa; and GAPDH – 35KDa. The data were analysed with analysis of variance. *Significant difference (p < 0.05) between basal and agonist or LPS treatment. #Significant difference (p < 0.05) between HFD-O and SC-O.

Next, we evaluated the impact of central m1mAChR activation with McN-A-343 on the macrophage profile of isolated bone marrow cells (**Figure 7**). We noted a decrease in IL1-β protein expression in the culture medium in both groups following treatment with the m1mAChR agonist (**Figure 7A**). We also assessed *Tnf*, *Il1b*, *Il10*, *Il6*, *Nos2*, and *Arg1* mRNA expression (**Figure 7B**). Specifically, *Il1b*, *Il6*, and *Nos2* mRNA expression was significantly reduced upon administration of the agonist McN-A-343. Conversely, McN-A-343 administration increased *Il10* mRNA expression. Interestingly, *Il10* mRNA expression increased in both groups upon agonist treatment. Furthermore, there was decreased *Tnf* and *Il6* mRNA in HFD-O compared with SC-O. *Nos2* mRNA expression, indicative of the inflammatory profile, was decreased in HFD-O, and the agonist treatment further decreased *Nos2* mRNA expression in both groups. Conversely, *Arg1* mRNA expression, a marker of the anti-inflammatory profile, was increased in HFD-O (**Figure 7B**). Notably, phosphorylation of STAT3, a protein downstream in the cholinergic anti-inflammatory pathway, was increased in both groups following treatment with the m1mAChR agonist (**Figures 7C, D**).

## 4 Discussion

Obesity is widely acknowledged for its association with numerous comorbidities (35–37). Surprisingly, it seems to bestow a certain degree of protection against sepsis. Epidemiological data indicate that individuals with obesity exhibit a heightened likelihood of survival during clinical systemic inflammatory responses (1, 2). This intriguing phenomenon is commonly referred to as the ‘obesity paradox’ (3, 4, 6, 38), yet the actual protective mechanisms in the context of sepsis continue to be a subject of debate (5). An essential factor to consider in this analysis is the programming process during the intrauterine and lactation period, as per the Developmental Origins of Health and Disease (DOHaD) concept. However, the precise impact of maternal obesity on the inflammatory response of offspring during sepsis remains an unresolved question. What role does maternal obesity play in shaping the inflammatory response in the context of sepsis?

Several studies have demonstrated that offspring of obese dams exhibit metabolic impairments following inflammatory challenges (39–41). Sepsis is a complex disorder that is widely recognized as a leading cause of high mortality rates (42–44). It is characterized by an initial systemic inflammatory response syndrome (SIRS), followed by a counter-regulatory anti-inflammatory response syndrome (CARS) (45, 46). The prognosis for sepsis is closely related to the balance between pro- and anti-inflammatory responses.

We found that HFD-O exhibit enhanced resistance to death in LPS-induced sepsis. They also demonstrate increased expression of hypothalamic m1mAChR and reduced levels of inflammatory markers in the serum following LPS administration. Notably, inhibition of central m1mAChR completely abolishes the protective effect against sepsis mortality in HFD-O. Both HFD-O and SC-O show the onset of sepsis pathogenesis following LPS administration. However, HFD-O displays a quicker recovery compared to SC-O, indicating a more effective counter-regulatory anti-inflammatory response. Central m1mAChR appears to play a protective role against sepsis in HFD-O, potentially associated with attenuated sepsis-induced immune and metabolic dysregulation, as suggested by previous studies investigating the role of this receptor in the prevention of endotoxemia (21, 47). In contrast, a previous study demonstrated that knockout mice lacking m1mAChR exhibit higher perioperative mortality following invasive surgery to remove the adrenal glands (48).

The expression of cholinergic receptors appears to be regulated through post-transcriptional mechanisms. Zaghloul and colleagues (49) demonstrated a decrease in m1mAChR expression in the central nervous system of mice with CLP-induced sepsis. Additionally, a previous study from our group revealed that short-term HFD consumption reduces hypothalamic α7nAChR expression and increases mortality in a model of sepsis induced by CLP surgery (21). Interestingly, HFD-O did not show any modifications in hypothalamic α7nAChR expression but exhibited a significant increase in m1mAChR expression. These studies suggest that modulation of cholinergic receptor expression is a dynamic process that can be influenced by inflammatory conditions.

Epigenetic mechanisms during developmental phases, such as pregnancy and lactation, have been associated with improved prognostics in sepsis (50). Epigenetics plays a significant role in various stages of sepsis, including pathogen–host interactions, immunosuppression, and the inflammatory response (51, 52). In the later stages of sepsis, anti-inflammatory cytokines are produced, contributing to the immune tolerance of the host. This phenomenon, referred to as immunologic memory, may be associated with protection against future infections (50, 53).

The liver plays a crucial role in both inflammatory and innate immune responses (54, 55). Furthermore, studies have demonstrated the significant role of the liver in the response to sepsis stages (56, 57). The liver is responsible for the secretion of acute phase proteins (APP), which are regulated by IL-6 levels and STAT3 activation (54, 58, 59). Sander et al. (55) demonstrated that the activation of APP, such as amyloid A, along with elevated CXCL1 levels, promote the mobilization, accumulation, and survival of myeloid cells in the liver. Interestingly, our study revealed that pharmacological activation of hypothalamic m1mAChR in HFD-O leads to increased phosphorylation of liver STAT3 and expression of α7nAChR compared with SC-O. Furthermore, HFD-O exhibit higher liver IL-6 expression in response to LPS compared with SC-O. It is worth noting that the JAK2/STAT3 pathway, as demonstrated by De Jonge and Ulloa (60), operates downstream of α7nAChR and can reduce the inflammatory response by inhibiting NF-κB and TNFα expression. Consequently, there is an enhanced anti-inflammatory response in HFD-O due to downregulation of inflammatory cytokine expression and the stimulation of APP secretion. This effect is achieved through activation of liver α7nAChR as well as STAT3 via IL-6 signalling.

During the acute phase of sepsis, immune cells such as macrophages, T and B lymphocytes, and neutrophils in lymphoid tissues, including the spleen and thymus, undergo activation. However, in the late phase of sepsis, these cells experience substantial apoptosis (61, 62). We found an increase in CD4+ T lymphocytes and a decrease in CD8^+^ cells in HFD-O compared with SC-O. Similarly, a study examining sepsis induced by CLP also reported reduced activity of CD4^+^ T lymphocytes. However, activation of the CAP significantly reverses the immunosuppressive state of CD4^+^ T lymphocytes (63). Given that immune cells undergo apoptosis as sepsis progresses, the elevated levels of lymphocytes in the spleen prior to sepsis infection could potentially confer protection against the pathogenesis of sepsis.

CD4^+^ lymphocytes have the capacity to differentiate into various phenotypes, including Th1, Th2, Th17, and Th22. Th17 differentiation is initiated by the secretion of TGF-β, IL6, and IL1β, which induce the activation of RORγt, a transcription factor associated with Th17 differentiation (64). Lymphocyte CD4^+^ Th17 cells can exhibit pathogenic characteristics and induce an inflammatory response (64–66). On the other hand, the differentiation of lymphocyte CD4^+^ Th22 cells can be regulated by Th17 cells and the levels of IL-17 and IL-22. Studies have indicated that elevated levels of IL-6 contribute to the differentiation of naive CD4^+^ T cells into Th22 cells (67, 68). Our findings indicate that TGF-β and IL-17 levels are reduced in the spleen of HFD-O, while IL-22 and IL-6 levels are increased. As a result, lymphocytes in the spleen appear to differentiate into the Th22 phenotype rather than the Th17 phenotype. Th22 cells are associated with anti-inflammatory responses and play a role in promoting the innate immune defence against infections (69, 70). Furthermore, spleen macrophages and bone marrow–derived macrophages in HFD-O appear to exhibit an anti-inflammatory phenotype, even when confronted with an inflammatory challenge such as LPS. Boomer et al. (71) highlighted the crucial role of spleen macrophages in the anti-inflammatory response during sepsis. These macrophages are responsible for reducing cytokine levels, including IFNγ, TNF, IL-6, and IL-10, in patients with sepsis. Moreover, in sepsis survivors, there is an increase in myeloid progenitor cells, and trained immunity leads to the reprogramming of naïve bone marrow monocytes (72, 73).

In conclusion, our data provide evidence that HFD-O exhibit partial protection against LPS-induced sepsis. This protective effect appears to be mediated through upregulation of central m1mAChR. While we have identified this central muscarinic receptor as the primary activator of the CAP, we did not investigate the role of central α7nAChR in this process, as suggested by Ren and colleagues (63). However, we did not observe an increase in central α7nAChR expression in HFD-O compared with SC-O. Additional studies are required to fully understand the mechanism underlying the modulation of m1mAChR expression and its role in protecting offspring against sepsis mortality.

Although the results presented in this study suggest that maternal obesity brought advantages to the offspring in terms of the anti-inflammatory response, we need to be cautious in assimilating this information. It is widely known that other physiological processes are affected by maternal obesity. Therefore, it is essential to monitor the development of these offspring and the maintenance of this characteristic of the immune system. The use of anti-inflammatory therapies, although promising in these cases, needs to be used in the correct stages of sepsis evolution.

As limitations of study, we utilized an LPS model for inducing sepsis instead of the recommended CLP surgery. Our decision was influenced by the age of the involved offspring, which were 28 days old. We were concerned about the size of mice for conducting this highly impactful surgery, as it might potentially yield false positive results. Furthermore, while we evaluated IL17 and IL22 levels using qPCR, performing cytometry analysis of lymphocytes in the spleen might have provided more comprehensive insights. Our study assessed changes in the immune response in the offspring at 28 days of age. At this age, we cannot dismiss the contribution of maternal milk to the results shown. A study involving adult mice could provide us with information regarding the persistence of these alterations in the inflammatory response.

Furthermore, new studies need to be conducted to identify nutritional components and/or inflammatory factors that may act in the development of the immune system. Additionally, in our study, we did not investigate whether the programming of the inflammatory response occurred during the gestation or lactation period. However, this study demonstrates that the inflammatory response can be programmed in such a way as to provide individuals with greater protection in situations of exposure to infectious agents and may have been an important mechanism of evolutionary adaptation for many species.

## Data availability statement

The original contributions presented in the study are included in the article/supplementary material. Further inquiries can be directed to the corresponding author.

## Ethics statement

The animal study was approved by State University of Campinas Ethics Committee (Protocol 5733-1). The study was conducted in accordance with the local legislation and institutional requirements.

## Author contributions

SC: Conceptualization, Formal analysis, Investigation, Methodology, Validation, Writing – original draft. WC: Formal analysis, Investigation, Methodology, Validation, Writing – original draft. PL: Formal analysis, Investigation, Methodology, Validation, Writing – original draft. IS: Formal analysis, Investigation, Methodology, Validation, Writing – original draft. BB: Formal analysis, Investigation, Methodology, Writing – original draft. LI-S: Data curation, Formal analysis, Resources, Supervision, Writing – review & editing. AT: Funding acquisition, Methodology, Resources, Supervision, Writing – review & editing. MM: Methodology, Resources, Writing – review & editing. HR: Formal analysis, Investigation, Methodology, Resources, Writing – review & editing. MD: Funding acquisition, Resources, Writing – review & editing. MR: Funding acquisition, Resources, Writing – review & editing. MT: Conceptualization, Funding acquisition, Project administration, Resources, Supervision, Writing – review & editing.
